# Traditional Small-Size *Citrus* from Taiwan: Essential Oils, Bioactive Compounds and Antioxidant Capacity

**DOI:** 10.3390/medicines4020028

**Published:** 2017-05-08

**Authors:** Min-Hung Chen, Kai-Min Yang, Tzou-Chi Huang, Mei-Li Wu

**Affiliations:** 1 Department of Food Science, National Pingtung University of Science & Technology, Pingtung 90090, Taiwan; e933623366@yahoo.com.tw (M.-H.C.); tchuang@mail.npust.edu.tw (T.-C.H.); 2 Department of Food Science and Biotechnology, National Chung Hsing University, 250 Kuokuang Road, Taichung 40227, Taiwan; a9241128@gamil.com

**Keywords:** calamondin, kumquat, essential oils, flavonoids, antioxidant

## Abstract

**Background:** The calamondin (*Citrus microcarpa* Bunge) and the kumquat (*Fortunella crassifolia* Swingle) are two small-size citrus fruits that have traditionally been consumed in Taiwan; however, there has been a lack of scientific research regarding the active compounds and functionalities of these fruits. **Methods:** Analysis of volatile composition of essential oil and phytosterol was carried out using Gas Chromatography–Mass Spectrometry (GC-MS). Flavonoid and limonoid were analyzed by High Performance Liquid Chromatography (HPLC). Moreover, antioxidant capacity from their essential oils and extracts were assessed in vitro. **Results:** The compositions of the essential oils of both fruits were identified, with the results showing that the calamondin and kumquat contain identified 43 and 44 volatile compounds, respectively. In addition, oxygenated compounds of volatiles accounted for 4.25% and 2.04%, respectively, consistent with the fact that oxygenated compounds are generally found in high content in citrus fruits. In terms of flavonoids, the calamondin exhibited higher content than the kumquat, with disomin-based flavonoids being predominant; on the other hand, phytosterol content of kumquat was higher than that of calamondin, with amyrin being the dominant phytosterol. Both of them contain high amounts of limonoids. The ethanol extracts and essential oils of small-sized citrus fruits have been shown to have antioxidant effects, with those effects being closely related to the flavonoid content of the fruit in question. **Conclusions:** The present study also reviewed antioxidant activity in terms of specific bioactive compounds in order to find the underlying biological activity of both fruits. The calamondin and kumquat have antioxidant effects, which are in turn very important for the prevention of chronic diseases.

## 1. Introduction

**Figure 1:**
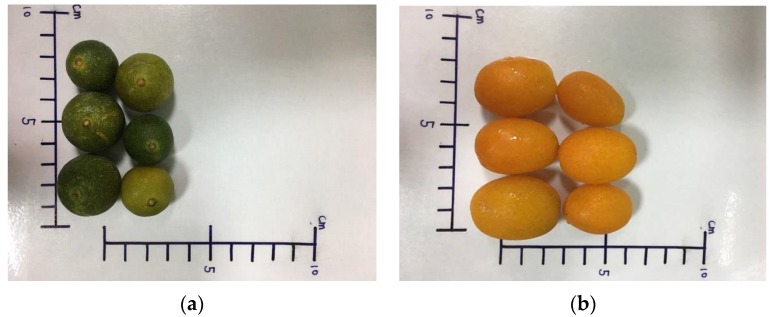
The morphological deviation of the calamondin (**a**) and kumquat (**b**) grown in the field.

Citrus fruits constitute a great proportion of fruit tree crops grown throughout the world, with fresh citrus fruits being exported or sold in local markets, and also used for processing. The taxonomic classification of the various species in the Citrus genus is complex and diverse. Citrus species fall under the Rutaceae family and its Aurantioideae subfamily, which is comprised of 33 well-known and thoroughly described genera and 203 species [1]. Additionally, the existence of many natural and artificial hybrids have resulted in new edible cultivars, which collectively constitute important varieties in terms of their wide range of uses, markets, growing conditions, and climatic zones [1,2]. The harvest times for the kumquat and calamondin (*Citrus microcarpa* Buonge) are from November-February and July-January, respectively. These two fruits are the smallest of the true citrus fruits (Figure 1) and have several health benefits, including being low in calories from sugar. The two fruits are traditionally used as folk medicine in Asian countries to manage inflammation of the respiratory tract [3]. As fresh fruits, they are sour to the taste, but both are widely used in processed fruit products, such as pickled preserves and marmalade. When fresh calamondin fruit is used with hot water to make beverages, there is some concern as to the resulting modifications of flavonoids and essential oils of the fruit [3,4,5]. Meanwhile, the peel of the kumquat is thin and full of flavonoids, and is edible along with the fruit flesh. As such, the flavonoid composition and biological activity of the kumquat are also subjects of some interest [3,6,7]. Flavonoid has strong antioxidant and radical scavenging activity which appears to be associated with reduced risk for certain chronic diseases, the prevention of cardiovascular disorders and cancers [8,9].

The possible beneficial effects of citrus fruits are due to the micronutrients (included ascorbic acid, dietary fiber, potassium and folate), functional ingredients, antioxidant nutraceuticals, and phytochemical substances that they contain [9]. These components of the fruits, especially when ingested daily, have exhibited various potentials for modulating human metabolism in a manner that may aid in the prevention of chronic and degenerative diseases [9]. Consequently, a large number of studies are being carried out on an ongoing basis on the thousands of phytochemicals that may have important physiological effects [3,9,10].

Of the citrus fruits grown by producers worldwide, about 30% of the total crop is processed to obtain various products, mainly juices [1,11]. There have been several reports on the relationship between processing treatments and the antioxidant compounds in citrus by-products that have indicated that regardless of the specific method of processing used, the phytochemical contents of such products are lower than those of whole citrus fruits [11,12]. Peel oils and pectins are important citrus by-products that are widely used in products intended for human consumption, including foods, pharmaceuticals, and cosmetics. Citrus flavedo extracts represent a significant source of flavonoids and carotenoids, which have potentially prophylactic properties that may make them of use in the development of functional foods, meaning foods with various health-promoting properties such as being antiatherogenic, anti-inflammatory, antitumor, inhibitory against blood clots, and high in antioxidant activity [9,10,11,12].

The kumquat and calamondin are part of the ethnobotany of Taiwan, but their biological activities have received only limited attention. As such, further analysis of their citrus quality, nutritional characteristics, and purity is important for the purposes of industrial applications utilizing the two fruits. More specifically, this study analyzed samples of the two fruits in order to determine their composition of essential oil, phytosterols, flavonoid, limonoids and their antioxidant activities.

## 2. Materials and Methods 

### 2.1. Plant Materials and Sample Preparation

Calamondin (*Citrus microcarpa* Buonge) and kumquat (*Fortunella crassifolia* Swingle) fruits were harvested from local farmland in Taichung in December 2016. Citrus fruits were dried by used oven-dried (<50 °C) and milled in a grinder (IKA-Werke GmbH & Co. KG, Staufen, Germany) to produce 0.8 millimeter-sized powder. Their powder was then stored at −18 °C ready for use to analysis bioactive compounds.

### 2.2. Chemical Standards and Reagents

2,2′-Azinobis(3-ethylbenzothiazolin-6-sulfonic Acid) (ABTS), 1,1-diphenyl-2-picrylhydrazyl (DPPH), 6-hydroxy-2,5,7,8-tetramethylchroman-2-carboxylic acid (Trolox), diosmin, hesperetin, naringin, quercetin, and hesperdin were commercially available via Sigma Chemical Co. (St. Louis, MO, USA). The other analytical grade chemicals mentioned were purchased from CHEMICAL CO., LTD (Miaoli, Taiwan).

### 2.3. Steam Distillation of Essential Oils

Fresh citrus fruits (300 g) were homogenized (Waring Blender Model HGB7WTS3, Waring Co., Torrington, CT, USA) for 2 min with 1000 mL purified water and placed into a 5 L round-bottomed flask. The homogenate was then steam distilled for 3 h in order to obtain the corresponding essential oils, which were then stored in the dark at −20 °C.

### 2.4. Gas Chromatography–Mass Spectrometry (GC-MS) Analysis

The volatile compounds were identified by Agilent 6890 GC (Agilent, Palo Alto, CA, USA) equipped with DB-1 fused-silica capillary column (60 m × 0.25 mm × 0.25 μm, Agilent, Palo Alto, CA, USA), which was coupled to an Agilent 5973 N MSD detector (Agilent, Palo Alto, CA, USA). The temperature of injector was set at 250 °C with carrier gas (helium) flow at a 1 mL/min rate. The ionization potential was set 70 eV at 230 °C. The constituents’ spectra were compared to the published record in a mass spectral library (Wiley 7n,). Additionally, a n-alkanes (C_5_~C_25_) reference mixture (St. Louis, MO, USA) was used to calculate the retention indices (RI), that with those of authentic standards or those in the published literature.

### 2.5. High Performance Liquid Chromatography (HPLC) Analysis of Flavonoids ana Limonoid 

The flavonoid standards used included: naringin, hesperidin, diosmin, quercetin, and hesperetin, which were prepared of methanol. The sample was conducted by reflux extraction with methanol for 2 h. The quantitative determination method of flavonoid composition was described previously [13]. The analysis method of limonoid quantitative determination was also described previously, as limonoid standards were dissolved in acetonitrile [14]. A 20 μL aliquots of filtrate were injected into a injection port and separated by an HPLC system (L-2130 pump and L-2400 UV detector, Hitachi, Tokyo, Japan) attached to RP-18GP250 column Mightysil (*l* = 250 mm; i.d. = 4.6 mm; thickness = 0.32 μm; Kanto Chemical Co., Inc., Tokyo, Japan). The calibration curves of each standard were established by plotting the peak area vs. corresponding concentration, respectively.

### 2.6. GC Analysis of Phytosterol Composition 

The sample was conducted by reflux extraction with hexane for 2 h. The procedures were reported previously: [15] Agilent 6890 GC (Agilent, Palo Alto, CA, USA) was equipped with a DB-1 fused-silica capillary column (Agilent, Palo Alto, CA, USA). The temperature of injector was set at 250 °C with nitrogen flow at a 1 mL/min rate. GC was also used to analyze the phytosterol derivative extracts with a 1:30 split ratio injection at 260 °C. The temperature of initial column was held at 50 °C for 0.5 min, and then it increased at a rate of 20 °C/min to 320 °C and maintained for a another 10 min with a flow rate of 1.4 mL/min. The FID was set at 320 °C. The contents of phytosterol were determined by the normalization method, as 5α-cholesterol was used as an internal standard to quantitation.

### 2.7. Antioxidant Capacity Assay

For the purpose of evaluating the antioxidant activity of small-sized citrus fruit samples, the biochemical methods of total phenolic, total flavonoid, DPPH, and ABTS radical-scavenging assays were used. The tests were carried out in triplicate. Kumquat and calamondin (1 g) were extracted with 15 mL ethanol at room temperature for 2 h and centrifuged at 3000× *g* for 15 min. Total phenolic compound as gallic acid equivalents mg/g of dry peel weight using Foline-Ciocalteu reagent, and total flavonoid content as quercetin equivalents mg/g of dry peel weight using AlCl_3_ colorimetric method [16]. The DPPH radical-scavenging activity was detected according to previous research [17]. Kumquat and calamondin (ethanol extracts and essential oil extracts) were dissolved in a DPPH-radical-contained ethanol solution (0.2 mM). Shaking and incubating for 30 min, the sample was measured by UV absorbance at 517 nm. The scavenging activity of ABTS radicals was modified according to previous research [18]. ABTS^+^ were produced by having ABTS solution (7 mM) reacted with potassium persulphate (1.4 mM), and allow the ABTS^+^ solution standing in the dark for 16 h. Upon using, the ABTS^+^ solution was diluted with methanol to a suitable absorbance of 0.80 ± 0.05 at 734 nm. To the solution mixed with ABTS^+^ was added samples of ethanol extracts and essential oil extracts. After reacting for 5 min at room temperature, the absorbance at 734 nm was measured again. The DPPH and ABTS scavenging expressed as mg trolox equivalent μg/g of dry peel weight. 

### 2.8. Statistical Analysis 

All experiment was performed in triplicate and all data were expressed in a form of mean ± standard deviation of the mean (SD). Analysis of variance (ANOVA), with SPSS 10.0 (SPSS, Chicago, IL, USA), was used to analyze data obtained in the same group. In order to test the significance of the differences between paired means, Duncan’s multiple range test was used. A confidence level of *p* < 0.05 was applied to judge the significance of each difference.

## 3. Results and Discussion

### 3.1. Analysis of Volatile Essential Oils

Essential oils are produced by cells within the rind of a citrus fruit. In the presence of air and heat, these oils evaporate; thus, they were termed as volatile oils [19]. In this study, it was found that the essential oil extraction rates via steam distillation for the calamondin and kumquat were 0.75% and 0.71% (*w*/*w*), respectively. GC-MS was used to identify the volatile components of essential oils. Table 1 lists the retention indices and relative area percentages. Fifty-eight volatile compounds were totally identified across the different samples tested. Grouped according to chemical structure, these compounds consisted of monoterpenes (11), sesquiterpenes (13), aldehydes (11), alcohols (16), esters (6), and a ketone.

We detected 43 volatile compounds in the essential oil obtained from the calamondin samples. Of those compounds, the content of limonene was the highest (87.52%), followed by those of β-myrcene (4.75%), α-pinene (1.41%), α-terpineol (1.51%), α-terpinolene (0.68%), and geranyl acetate (0.40%). We detected 44 volatile compounds in the essential oil obtained from the kumquat samples. Of those compounds, the content of limonene was the highest (89.60%), followed by those of β-myrcene (4.42%), α-pinene (1.28%), germacrene-D (1.16%), α-terpineol (0.55%), and geranyl acetate (0.36%). The terpene compounds of essential oils do not contribute to their flavor and fragrance. However, these terpene compounds were found to be the main components of the oleoresin in the tested samples, accounting for 95.75% of the essential oil in the calamondin samples and 97.96% of the essential oil in the kumquat samples (Table 1). 

Because terpenes themselves are light and heat sensitive, they are prone to deterioration during storage. Heat stress induces the oxidative degradation of limonene, resulting in the formation of monoterpene hydrocarbons or oxygenated monoterpenes [5,6,20]. The terpineol and carvone components are formed by the oxidative degradation of limonene and are well known for their contribution to the loss of flavor and quality of citrus juices. Meanwhile, the floral notes of linalool have been identified as important contributors to the aroma of citrus fruits [21,22]. Linalool itself has a floral smell, and acts synergistically with other components, strengthening the overall floral aroma. Limonene is also essential to the background aroma, and aliphatic aldehydes, which are among the active compounds of citrus, express a sweet waxed aroma and citrus peel-like odor [23]. Deterpenation of essential oil in distillation increases flavor and oxidation stability; however, it causes changes in the quantity of volatile compounds. A 10-fold condensation of the orange oil causes a decreased (97.37% to 41.06%) in the terpene compounds and an increased (1.78% to 45.74%) in the oxygenated compound [24].

### 3.2. Analysis of Bioactive Compound

Flavonoids from various citrus species have numerous biological properties, particularly antioxidant and anti-inflammatory. For example, naringin, neohesperidin, and neoeriocitrin are mainly exist in grapefruit and bitter orange juices, while hesperidin, narirutin, and didymin are exist in orange, mandarin, and lemon juices [25]. As shown in Table 2, the flavonoids found in the calamondin samples were diosmin (5.99 μg/g), hesperetin (3.31 μg/g), naringin (1.66 μg/g), quercetin (0.53 μg/g), and hesperidin (0.42 μg/g). The flavonoids found in the kumquat samples were naringin (0.52 μg/g), diosmin (0.35 μg/g), hesperetin (0.05 μg/g), and hesperdin (0.01 μg/g). The flavedo of citrus fruits is high in flavonoids, so the flavonoid content of the kumquat is relatively low due to its thin flavedo. At present, it is known that flavonoids are heterogeneous in terms of their different molecular structures, but there is a scarcity of data on the bioavailability of the various flavonoids [25,26,27]. Flavonoids are ubiquitous in plant foods, and their toxicity is very low in animals. Nonetheless, as a precaution, doses of less than 1 mg per adult per day have been recommended for humans [27].

Several clinical studies have been conducted to check the safety of foods enriched with phytosterols. According to hematology and clinical chemistry studies, there is no evidence that phytosterols have significant toxic effects, and they have been found to be neither genotoxic or teratogenic [28,29]. Naturally occurring phytosterols, which are taken in as part of habitual dietary intake at a range of 150–450 mg/day, are negatively correlated with cholesterol absorption [29]. In this study, the total phytosterol content of the calamondin samples was found to be 28.27 μg/g, while that of the kumquat samples was found to be 12.56 μg/g (Table 2). The analysis results identified five kinds of phytosterols in these small-sized citrus fruits. The calamondin samples contained campesterol (4.43 μg/g), stigmasterol (4.52 μg/g), sitosterol (1.57 μg/g), and amyrin (2.07 μg/g). The kumquat samples contained campesterol (1.02 μg/g), stigmasterol (1.33 μg/g), β-sitosterol (7.04 μg/g), amyrin (10.45 μg/g), and lupenone (8.43 μg/g). Phytosterols, which are generally dominated by the structure of cholesterol with one or two extra carbon atoms in the side chain, can inhibit the absorption of endogenous cholesterol, leading to their cholesterol-lowering effect [28]. 

Limonoids, which are water-insoluble and cause a bitter taste, have been found to be present in citrus fruits in amounts of 0~95.46 mg/100 g [30]. The results of this study of small-sized citrus established that the samples were rich in limonoids, with calamondin and kumquat samples respectively containing 1.85 and 1.44 μg/g of limonin, and the nomilin contents being 0.19 and 0.16 μg/g, respectively. Limonoids are highly oxygenated triterpenoids without a hydrogen atom available to donate to any reactions, leading to their poor free radical scavenging ability in vitro [14]. A previous study showed that limonoids could have a protective effect against low-density lipoprotein (LDL) oxidation. Limonoids are human health promoters, and have many pharmacological properties, including anticancer, antioxidant, antibacterial, and antifungal properties [14,30].

### 3.3. Antioxidant Property

As expected, the non-volatile resins were found to be low in free radical scavenging activities in both DPPH and ABTS tests. In order to characterize the major antioxidant effective compounds in these two small-sized citrus fruits, extractions of calamondin and kumquat with 95% ethanol were carried out, and the resulting extracts were then subjected to an analysis of their antioxidant activities and phenolic compounds. The results showed that with regard to the antioxidants in the calamondin and kumquat samples, the total phenolic contents were 5.77 and 2.29 GAE mg/g, respectively, and the total flavonoid contents were 2.71 and 1.36 QE mg/g, respectively. In terms of the antioxidant capacity of the calamondin and kumquat samples, the DPPH radical clearing capacities were 1.15 and 0.82 mg Trolox/g, respectively, and the ABTS radical clearing capacities were 3.83 and 0.95 mg Trolox/g, respectively (Table 3). The kumquat samples had higher antioxidant capacity than that of calamondin samples due to their richer total phenolic and total flavonoid contents. According to previous reference the juice of citrus species in china, showed that total phenolic were 0.75~1.55 GAE mg/L and DPPH scavenging 23.69~61.62% [31].

In this study, the DPPH and ABTS radical clearing capacity in the essential oils of Kumquat and calamondin were found to be in the range of 29.38~54.63 μg Trolox/mL, and 85.21~115.64 μg Trolox/mL, respectively (Table 3). Previous reports have mentioned that polar extracts of citrus fruits, such as ethyl acetate extract, ethanol extract and supercritical fluid extract, exhibit better radical-scavenging activity than essential oil extracts [4,16,32,33]. However, it is difficult to attribute the antioxidant effects of a total essential oil to just one or a few active compounds, as both minor and major compounds should make significant contributions to an oil’s activity. The antioxidant potential of volatile compounds is well established as phenolics and secondary metabolites with conjugated double bonds [34]. All citrus essential oils have radical-scavenging activity, the efficacy of which seems to depend on the content of such compounds as γ-terpinene, terpinolene, and citral, all of which exhibit notable activity [35,36]. According to the previous literature, carvacrol, which is found in the essential oils of thymol and origanum, has DPPH-scavenging capacity [35]. Moreover, in essential oils of citrus fruits, citral has been found to have greater oxidation stability than limonene and linalool [37]. Flavonoids are powerful antioxidants against free radicals which could form stable quinonemethide when combined with DPPH, leading to proton transferal [38]. The structure-antioxidant activity relationships of citrus flavonoid subclasses are highly dependent on the structures as well as the substituents of the heterocyclic and B rings [39]. More specifically, the major factors for radical-scavenging capability lie on (i) the presence of a catechol group in ring B, which is considered better electrondonating properties and a preferred radical target, and (ii) a 2,3-double bond conjugated with the 4-oxo group is accounted for effective electron delocalization [40]. Naringin, hesperidin, diosmin, and hesperitin have been determined to be the major active components responsible for the antioxidant activity of citrus fruits, and of those, hesperitin has the greatest radical-scavenging capacity due to its structure consisting of aglycones [39,40]. Other works in the literature indicate the antioxidant capacity of citrus bioactive compound, and have shown that the DPPH radical clearing capacities had the following relation: rutin > naringin > naringenin > limonin [41].

## 4. Conclusions

The results of this study confirm that the calamondin and kumquat have antioxidant effects, which are in turn very important for the prevention of chronic diseases. At the same time, many physiological effects do not depend on a single compound; rather, the additive effects of various compounds are greater than the effect of any single compound alone, so the compositions of the two fruits in terms of essential oils, flavonoids, and phytosterols were investigated in this study. The results of the present study not only provide information regarding the nutritive value of the kumquat and calamondin when they are consumed as fresh fruits, but also provide a basis for the evaluation of their various by-products.

## Figures and Tables

**Table 1:** Volatile constituents (%) of small-size citrus.

Compounds ^3^	RI ^2^	Formula	Composition (%) ^1^	
			Calamondin	Kumquat
Monoterpene				
α-pinene	934	C_10_H_16_	1.41	1.28
camphene	948	C_10_H_16_	0.04	0.01
sabinene	968	C_10_H_16_	n.d. ^4^	0.05
β-pinene	974	C_10_H_16_	0.39	0.03
β-myrcene	983	C_10_H_16_	4.75	4.42
α-phellandrene	997	C_10_H_16_	0.25	0.18
α-terpinene	1011	C_10_H_16_	0.24	0.02
limonene	1036	C_10_H_16_	87.52	89.60
β-ocimene	1045	C_10_H_16_	0.11	0.03
γ-terpinene	1055	C_10_H_16_	0.26	0.12
α-terpinolene	1082	C_10_H_16_	0.68	0.17
Sesquiterpene				
δ-elemene	1338	C_15_H_24_	0.06	0.15
α-copaene	1379	C_15_H_24_	n.d.	0.04
β-elemene	1389	C_15_H_24_	n.d.	0.08
β-caryophyllene	1422	C_15_H_24_	0.01	0.03
α-caryophyllene	1455	C_15_H_24_	0.01	0.03
α-muurolene	1477	C_15_H_24_	n.d.	0.04
germacrene-D	1481	C_15_H_24_	n.d.	1.16
bicyclogermacrene	1494	C_15_H_24_	n.d.	0.28
δ-cadinene	1501	C_15_H_24_	n.d.	0.04
γ-cadinene	1509	C_15_H_24_	n.d.	0.02
β-cadinene	1516	C_15_H_24_	n.d.	0.13
α-gurjunene	1532	C_15_H_24_	n.d.	0.02
germacrene-B	1556	C_15_H_24_	0.01	0.03
Esters				
heptyl acetate	1092	C_9_H_18_O_2_	0.01	n.d.
octyl acetate	1191	C_10_H_20_O_2_	0.05	0.13
nonyl acetate	1289	C_11_H_22_O_2_	0.01	n.d.
citronellyl acetate	1331	C_12_H_22_O_2_	n.d.	0.08
neryl acetate	1340	C_12_H_20_O_2_	0.07	0.05
geranyl acetate	1357	C_12_H_20_O_2_	0.40	0.36
Ketones				
carvone	1217	C_10_H_14_O	0.04	0.03
Alcohols				
3-hexene-1-ol	837	C_6_H_12_O	0.02	0.01
linalool	1085	C_10_H_18_O	0.19	0.13
myrcenol	1098	C_10_H_18_O	0.02	n.d.
α-fenchol	1100	C_10_H_18_O	0.03	n.d.
β-terpineol	1129	C_10_H_18_O	0.42	0.19
4-terpinenol	1165	C_10_H_18_O	0.26	0.15
α-terpineol	1176	C_10_H_18_O	1.51	0.55
carveol	1198	C_10_H_16_O	0.02	0.06
geraniol	1234	C_10_H_18_O	n.d.	0.01
nerolidol	1546	C_15_H_26_O	n.d.	0.02
ledol	1579	C_15_H_26_O	n.d.	0.05
10-epi-γ-eudesmol	1622	C_15_H_26_O	0.08	0.01
muurolol	1630	C_15_H_26_O	0.02	0.04
β-eudesmol	1641	C_15_H_26_O	0.08	n.d.
α-cadinol	1642	C_15_H_26_O	n.d.	0.07
α-eudesmol	1646	C_15_H_26_O	0.03	n.d.
Aldehydes				
hexanal	774	C_6_H_12_O	0.01	n.d.
2-hexenal	825	C_6_H_10_O	0.06	0.02
heptanal	878	C_7_H_14_O	0.01	n.d.
octanal	980	C_8_H_16_O	0.19	n.d.
decanal	1184	C_10_H_20_O	0.39	0.08
2-decenal	1238	C_10_H_18_O	0.06	n.d.
citral	1243	C_10_H_16_O	0.02	n.d.
perillal	1248	C_10_H_14_O	0.02	0.02
2,4-decadienal	1268	C_10_H_16_O	0.07	n.d.
undecanal	1285	C_11_H_22_O	0.10	n.d.
dodecanal	1386	C_12_H_24_O	0.06	n.d.
terpene compounds			95.75	97.96
oxygenated compounds			4.25	2.04

^1^ Each value is the mean of three replications; ^2^ RI: Retention index; ^3^ identified via comparison of the mass spectra with the RI; ^4^ n.d.: not detected.

**Table 2:** Flavonoids, phytosterols and limonoid composition of small-size citrus.

Compounds	Calamondin	Kumquat
flavonoids (μg/g dry base)		
naringin	1.66 ± 0.11 ^b^	0.52 ± 0.02 ^a^
hesperidin	0.42 ± 0.02 ^b^	0.05 ± 0.01 ^a^
diosmin	5.99 ± 0.36 ^b^	0.35 ± 0.03 ^a^
quercetin	0.53 ± 0.02 ^b^	n.d. ^1^
hesperitin	3.31 ± 0.05 ^b^	0.08 ± 0.01 ^a^
phytosterols (μg/g dry base)		
campesterol	4.43 ± 0.28 ^b^	1.02 ± 0.08 ^a^
stigmasterol	4.52 ± 0.32 ^b^	1.33 ± 0.11 ^a^
sitosterol	1.54 ± 0.75 ^a^	7.04 ± 0.52 ^b^
amyrin	2.07 ± 0.14 ^a^	10.45 ± 0.83 ^b^
lupenone	n.d.	8.43 ± 0.36 ^a^
limonoid (μg/g dry base)		
limonin	1.85 ± 0.11 ^b^	1.44 ± 0.08 ^a^
nomilin	0.19 ± 0.04 ^a^	0.16 ± 0.03 ^a^

Data presented are in mean ± SD (*n* = 3) which with different letters are significantly different at *p* < 0.05.; ^1^ n.d.: not detected.

**Table 3:** Assay for total phenol, total flavonoid, ABTS and DPPH scavenging abilities of small-size citrus.

Antioxidant Capacity	Calamondin	Kumquat
EtOH extracted of fruit dry base		
Total phenolic (GAE mg/g)	5.77 ± 0.34 ^b^	2.29 ± 0.15 ^a^
Total flavonoid (QE mg/g)	2.71 ± 0.15 ^b^	1.36 ± 0.07 ^a^
DPPH (Tr mg/g)	1.15 ± 0.06 ^b^	0.82 ± 0.02 ^a^
ABTS (Tr mg/g)	3.83 ± 0.08 ^b^	0.95 ± 0.03 ^a^
Essential oil		
DPPH (Tr ug/mL)	29.38 ± 0.62 ^a^	54.63 ± 0.83 ^b^
ABTS (Tr ug/mL)	85.21 ± 0.51 ^a^	115.6 ± 1.02 ^b^

Data presented are in mean ± SD (*n* = 3) which with different letters are significantly different at *p* < 0.05. GAE: gallic acid equivalents; QE: qucercetin equivalents; Tr: Trolox equivalents.
